# Phylogenomics of a rapid radiation: is chromosomal evolution linked to increased diversification in north american spiny lizards (Genus *Sceloporus*)?

**DOI:** 10.1186/s12862-016-0628-x

**Published:** 2016-03-22

**Authors:** Adam D. Leaché, Barbara L. Banbury, Charles W. Linkem, Adrián Nieto-Montes de Oca

**Affiliations:** Department of Biology, University of Washington, Seattle, Washington, 98195 USA; Burke Museum of Natural History and Culture, University of Washington, Seattle, Washington 98195 USA; Fred Hutchinson Cancer Research Center, 1100 Fairview Ave. N., Mail Stop M4-B402, Seattle, 98109 Washington USA; Departamento de Biología Evolutiva, Facultad de Ciencias, Universidad Nacional Autónoma de México, Ciudad Universitaria, 04510 México

**Keywords:** Gene tree, Lizards, Phrynosomatidae, Phylogenomics, Species tree, Systematics

## Abstract

**Background:**

Resolving the short phylogenetic branches that result from rapid evolutionary diversification often requires large numbers of loci. We collected targeted sequence capture data from 585 nuclear loci (541 ultraconserved elements and 44 protein-coding genes) to estimate the phylogenetic relationships among iguanian lizards in the North American genus *Sceloporus*. We tested for diversification rate shifts to determine if rapid radiation in the genus is correlated with chromosomal evolution.

**Results:**

The phylogenomic trees that we obtained for *Sceloporus* using concatenation and coalescent-based species tree inference provide strong support for the monophyly and interrelationships among nearly all major groups. The diversification analysis supported one rate shift on the *Sceloporus* phylogeny approximately 20–25 million years ago that is associated with the doubling of the speciation rate from 0.06 species/million years (Ma) to 0.15 species/Ma. The posterior probability for this rate shift occurring on the branch leading to the *Sceloporus* species groups exhibiting increased chromosomal diversity is high (posterior probability = 0.997).

**Conclusions:**

Despite high levels of gene tree discordance, we were able to estimate a phylogenomic tree for *Sceloporus* that solves some of the taxonomic problems caused by previous analyses of fewer loci. The taxonomic changes that we propose using this new phylogenomic tree help clarify the number and composition of the major species groups in the genus. Our study provides new evidence for a putative link between chromosomal evolution and the rapid divergence and radiation of *Sceloporus* across North America.

**Electronic supplementary material:**

The online version of this article (doi:10.1186/s12862-016-0628-x) contains supplementary material, which is available to authorized users.

## Background

Rapid radiations represent some of the most intriguing and well-studied biological systems. They also present some of the most difficult phylogenetic problems. The short time intervals separating the speciation events that occur during a rapid radiation leave few opportunities for molecular evolutionary changes to become established in the genome. This lack of phylogenetic information typically leads to large-scale gene tree discordance and a lack of resolution for the phylogenetic relationships [1]. Species involved in rapid radiations are typically partitioned into major clades with clear support from multiple sources of data, yet the interrelationships among the major clades are often ambiguous. This basic conundrum repeats itself across the Tree of Life (e.g., the root of life [2, 3], major bird orders [4, 5], Mammals [6, 7], and Neobatrachian frogs [8]). Attempting to resolve rapid radiations using a combination of large numbers of loci together with coalescent-based species tree inference methods [9–14] represents an important new direction in systematic biology this is expected to help resolve difficult phylogenetic problems.

There are at least three fundamental challenges confronting the resolution of rapid radiations using molecular genetic data: 1) quick bursts of speciation limit the opportunities for character changes to accumulate across the genome [1], 2) long-branch attraction artifacts during phylogeny estimation [15], and 3) incomplete lineage sorting [16]. Increasing the number of loci used to estimate the phylogeny can sometimes help alleviate the first problem [17–19]. However, depending on the method and the model, increasing the amount of data can be positively misleading when faced with long branch attraction and/or incomplete lineage sorting [15, 20, 21]. Overcoming these collective challenges, which are not mutually exclusive and are difficult to distinguish, requires the acquisition of large datasets composed of many independent loci together with the implementation of coalescent models of phylogenetic inference; however, analyzing large datasets is computationally demanding, and this problem is amplified when utilizing complex coalescent-based models. Our ability to generate sequence data is quickly outpacing our capacity to analyze genetic data under complex models such as the multispecies coalescent [22]. Coalescent methods that utilize gene trees instead of sequence data can dramatically decrease computation times [23], but this comes at the cost of information loss as uncertainty in the sequence data is not taken into account.

The phrynosomatid lizard genus *Sceloporus* is a diverse clade containing 90 + species with a broad distribution across North America [24]. Developing a robust phylogenetic framework for comparative studies of *Sceloporus* has been of interest for decades (reviewed by [24–30]). Previous phylogenetic studies of *Sceloporus* based on a few nuclear genes suggest that the group has experienced a period of rapid evolutionary diversification [27]. These successive and rapid speciation events have resulted in bursts of speciation that have impeded the inference of a fully-resolved and strongly supported phylogeny [25, 28, 29]. Differentiation in the fundamental number of chromosomes among species and species groups is hypothesized to be a primary factor responsible for driving the rapid radiation of *Sceloporus* [27, 31]. The genus is comprised of 19 species groups containing anywhere from one species (two of the species groups are monotypic) to 15 species (Table 1). Most of the polytypic species groups have been the focus of detailed phylogeographic and phylogenetic study, including the *formosus* group [32], *grammicus* group [33], *torquatus* and *poinsettii* groups [34, 35], *magister* group [36], *scalaris* group [37], *spinosus* group [38], *undulatus* group [39, 40], and the *variabilis* group [41]. These systematic studies have advanced our knowledge of the interrelationships within many species groups; however, resolving the phylogenetic relationships among the species groups has remained difficult [28, 29].

**Table 1:** Specimens included in the study

Species	Voucher	RAW reads	Clean pairs	idba contig	idba loci
*angustus* group (2/2)					
*Sceloporus angustus*	LACM 134741	3,788,618	3,457,044	1205	579
*Sceloporus grandaevus*	ROM 26215	1,342,322	1,134,076	700	521
*clarkii* group (2/2)					
*Sceloporus clarkii*	MVZ 245876	2,844,162	2,543,574	1223	579
*Sceloporus melanorhinus*	MZFC 7454	3,384,030	2,971,666	1298	580
*formosus* group (11/15)					
*Sceloporus acanthinus*	ANMO1932	3,734,696	3,350,382	1971	575
*Sceloporus adleri*	UWBM 6608	8,137,738	7,364,486	4460	576
*Sceloporus cryptus*	MZFC 7438	2,350,112	2,133,386	1036	577
*Sceloporus druckercolini*	JAC 25172	2,760,330	2,463,818	1357	559
*Sceloporus formosus*	RVT 76	3,354,056	2,960,696	1550	569
*Sceloporus formosus*	ANMO 1248	2,192,170	1,693,102	948	541
*Sceloporus formosus scitulus*	UWBM 6623	5,476,146	4,950,836	2243	577
*Sceloporus internasalis*	JAC 22552	4,393,164	3,792,716	2119	573
*Sceloporus lunaei*	not sampled	–	–	–	–
*Sceloporus lundelli*	not sampled	–	–	–	–
*Sceloporus malachiticus*	MVZ 263420	5,373,316	4,945,178	2673	578
*Sceloporus salvini*	not sampled	–	–	–	–
*Sceloporus smaragdinus*	unknown	1,891,310	1,461,336	938	546
*Sceloporus stejnegeri*	MZFC 7452	3,981,586	3,470,518	3492	383
*Sceloporus subpictus*	MZFC 8028	6,456,814	5,737,156	5349	572
*Sceloporus taeniocnemis*	MVZ 264322	1,892,698	1,423,322	771	247
*Sceloporus tanneri*	not sampled	–	–	–	–
*gadoviae* group (2/2)					
*Sceloporus gadoviae*	UWBM 7309	6,519,836	5,991,130	1835	577
*Sceloporus maculosus*	JAM 650	3,140,176	2,691,774	2339	339
*graciosus* group (3/3)					
*Sceloporus arenicolus*	ADL 047	2,637,380	2,266,994	946	579
*Sceloporus graciosus*	MVZ 240898	6,241,172	5,631,350	3413	576
*Sceloporus vandenburgianus*	TWR 430	4,961,440	4,386,238	1435	581
*grammicus* group (6/6)					
*Sceloporus anahuacus*	AMH684	3,986,550	3,219,532	1512	573
*Sceloporus asper*	JAC23686	7,225,354	4,490,778	2723	573
*Sceloporus grammicus*	UWBM 6585	4,406,008	3,910,096	2054	576
*Sceloporus grammicus microlepidotus*	UOGV2525	1,315,156	1,051,322	702	518
*Sceloporus heterolepis*	MZFC 8017	4,824,734	3,952,154	4777	344
*Sceloporus palaciosi*	UWBM 7313	10,419,276	9,501,996	2546	578
*Sceloporus shannonorum*	JADE 220	5,377,848	5,003,792	4205	563
*jalapae* group (2/2)					
*Sceloporus jalapae*	UWBM 7318	4,557,808	4,128,130	1228	575
*Sceloporus ochoterenae*	UWBM 6641	3,716,194	3,242,038	1449	579
*magister* group (6/6)					
*Sceloporus hunsakeri*	ADG 98	2,336,356	1,724,688	1168	580
*Sceloporus licki*	ADG 73	5,951,168	5,355,664	4806	554
*Sceloporus lineatulus*	unknown	8,289,082	7,293,688	7954	567
*Sceloporus magister cephaloflavus*	UWBM 7395	4,391,974	3,903,318	1457	578
*Sceloporus magister bimaculosus*	DGM 924	1,818,256	1,472,176	1015	549
*Sceloporus magister magister*	ROM 14488	8,938,692	8,446,102	5308	561
*Sceloporus magister uniformis*	DGM 474	11,528,354	10,328,510	8009	574
*Sceloporus magister uniformis*	MVZ 162077	4,856,264	3,818,418	2286	562
*Sceloporus orcutti*	UWBM 7654	5,061,334	4,346,088	1695	579
*Sceloporus orcutti*	RWM 798	3,769,420	2,886,214	1486	564
*Sceloporus zosteromus*	ADG 49	3,256,502	2,843,448	1274	577
*Sceloporus zosteromus*	ADG 74	14,267,908	12,650,332	5117	579
*megalepidurus* group (2/3)					
*Sceloporus halli*	not sampled	–	–	–	–
*Sceloporus megalepidurus*	MZFC 8026	4,443,820	3,916,852	3922	546
*Sceloporus pictus*	LCM 1149	3,581,154	3,310,488	2674	576
*merriami* group (1/1)					
*Sceloporus merriami*	LSUMZ 48844	3,786,064	3,181,240	1134	578
*poinsettii* group (10/12)					
*Sceloporus aureolus*	RVT 54	5,861,080	5,350,974	2627	566
*Sceloporus aureolus*	JAC 22409	5,310,894	4,798,000	2361	568
*Sceloporus cyanogenys*	FMQ 3250	3,588,644	3,028,816	3126	378
*Sceloporus cyanostictus*	unknown	2,276,708	1,830,554	1122	549
*Sceloporus dugesii*	UTAR 23955	3,147,022	2,445,678	1887	236
*Sceloporus macdougalli*	MZFC 7017	5,546,916	4,884,484	3456	559
*Sceloporus minor*	UOGV 1369	6,196,168	5,486,782	3883	564
*Sceloporus mucronatus*	UWBM 6636	4,831,958	4,464,672	2783	578
*Sceloporus oberon*	not sampled	–	–	–	–
*Sceloporus ornatus*	JAM 652	3,298,578	2,602,280	2533	311
*Sceloporus poinsettii*	LSUMZ 48847	3,518,050	3,088,022	3029	309
*Sceloporus serrifer*	UTAR 39870	3,255,154	2,896,292	2649	579
*Sceloporus sugillatus*	not sampled	–	–	–	–
*pyrocephalus* group (2/2)					
*Sceloporus nelsoni*	ANMO 3749	1,786,424	1,489,126	1380	578
*Sceloporus pyrocephalus*	unknown	2,960,302	1,701,102	723	567
*Sceloporus pyrocephalus*	UTAR 53473	2,510,072	1,987,732	1711	576
*scalaris* group (10/11)					
*Sceloporus aeneus*	RWB 769	3,950,636	3,201,546	1868	541
*Sceloporus aurantius*	RWB 1024	5,101,362	4,469,276	2943	544
*Sceloporus bicanthalis*	UWBM 7307	[45]	[45]	[45]	583
*Sceloporus brownorum*	RWB 6136	4,763,078	3,880,564	2676	559
*Sceloporus chaneyi*	RWB 6199	5,669,414	4,989,592	3018	543
*Sceloporus goldmani*	not sampled	–	–	–	–
*Sceloporus samcolemani*	JJW 698	7,011,786	6,414,192	6966	511
*Sceloporus samcolemani*	RWB 6263	1,252,600	1,174,070	865	509
*Sceloporus scalaris*	UWBM 6589	6,473,054	5,873,872	3253	575
*Sceloporus scalaris*	RWB 6247	3,654,246	3,176,608	2603	536
*Sceloporus slevini*	RWB 741	4,044,246	3,096,622	1329	526
*Sceloporus subniger*	RWB 686	2,348,238	2,073,796	3028	517
*Sceloporus subniger* "West"	RWB 645	7,806,116	7,173,556	1532	577
*Sceloporus unicanthalis*	JJ UANL 2/11	3,823,644	3,613,842	1651	558
*siniferus* group (3/4)					
*Sceloporus carinatus*	UWBM 6614	5,530,392	5,075,056	3202	577
*Sceloporus cupreus*	not sampled	–	–	–	–
*Sceloporus siniferus*	UWBM 6653	2,520,248	2,276,060	971	560
*Sceloporus siniferus*	MVZ 236299	4,928,056	4,507,972	1750	569
*Sceloporus squamosus*	UTAR 39846	2,220,578	1,990,330	1855	579
*spinosus* group (3/3)					
*Sceloporus edwardtaylori*	UWBM 6588	3,662,374	3,288,800	1769	578
*Sceloporus horridus*	MZFC 7458	2,687,744	2,205,246	2100	241
*Sceloporus spinosus*	UWBM 6672	3,349,020	3,069,336	1682	571
*torquatus* group (5/5)					
*Sceloporus bulleri*	FMQ 2815	4,561,554	4,287,202	2990	554
*Sceloporus insignis*	ANMO 1130	2,778,082	2,445,878	1275	554
*Sceloporus insignis*	unknown	8,364,794	7,792,166	6936	571
*Sceloporus jarrovii*	LSUMZ 48786	3,875,608	3,536,668	3359	535
*Sceloporus lineolateralis*	MZFC 6650	4,062,766	3,406,354	3365	264
*Sceloporus torquatus*	UWMB 6600	8,116,338	7,474,646	4694	576
*Sceloporus torquatus*	UOGV 2526	3,381,124	3,072,114	2917	578
*undulatus* group (10/10)					
*Sceloporus cautus*	MZFC 7414	8,275,890	7,070,754	1828	581
*Sceloporus consobrinus*	ADL105	1,417,172	1,185,840	773	534
*Sceloporus cowlesi*	AMNH 154059	4,275,658	3,889,018	2001	575
*Sceloporus exsul*	UWBM 6590	6,472,236	5,934,742	3189	574
*Sceloporus occidentalis*	UWBM 6281	[13]	[13]	[13]	540
*Sceloporus occidentalis*	MVZ 245697	2,319,900	2,137,418	1469	564
*Sceloporus olivaceus*	UWBM 7968	2,263,854	1,994,096	1088	580
*Sceloporus tristichus*	ADL189	5,483,728	5,010,016	2721	572
*Sceloporus undulatus*	ADL182	742,440	661,418	743	577
*Sceloporus virgatus*	MVZ 150112	2,818,968	2,451,942	905	575
*Sceloporus woodi*	MVZ 10643	1,430,400	1,277,004	870	574
*utiformis* group (0/1)					
*Sceloporus utiformis*	not sampled	–	–	–	–
*variabilis* group (6/7)					
*Sceloporus chrysostictus*	UTAR 53535	3,682,608	3,321,192	1536	572
*Sceloporus cozumelae*	not sampled	–	–	–	–
*Sceloporus couchii*	MZFC 6676	4,611,012	3,855,118	2613	394
*Sceloporus parvus*	MZFC 6664	4,973,960	4,489,574	5075	561
*Sceloporus smithi*	UWBM 6662	2,046,916	1,901,554	998	575
*Sceloporus teapensis*	UTAR 52778	4,352,968	3,926,160	3545	565
*Sceloporus variabilis*	UWBM 6678	6,819,682	6,201,808	2537	577
Sceloporinae outgroups					
*Petrosaurus thalassinus*	MVZ 161183	[13]	[13]	[13]	523
*Urosaurus ornatus*	UWBM 7587	[13]	[13]	[13]	577
*Uta stansburiana*	UWBM 7605	[13]	[13]	[13]	538
Callisaurini outgroups					
*Callisaurus draconoides*	MVZ 265543	[13]	[13]	[13]	575
*Callisaurus draconoides*	MVZ unknown	10,414,104	9,871,780	44,419	574
*Cophosaurus texanus*	UWBM 7347	[13]	[13]	[13]	573
*Holbrookia maculata*	UWBM 7362	[13]	[13]	[13]	573
*Uma notata*	SDSNH 76166	[13]	[13]	[13]	577
Phrynosomatini outgroups					
*Phrynosoma asio*	UWBM 7281	[45]	[45]	[45]	565
*Phrynosoma blainvillii*	CAS 200652	[45]	[45]	[45]	565
*Phrynosoma braconnieri*	UWBM7282	[45]	[45]	[45]	561
*Phrynosoma cerroense*	MVZ 161206	[45]	[45]	[45]	579
*Phrynosoma cornutum*	MVZ 238582	[45]	[45]	[45]	575
*Phrynosoma coronatum*	UABC 1007	[45]	[45]	[45]	364
*Phrynosoma ditmarsi*	RRM 2459	[45]	[45]	[45]	576
*Phrynosoma douglasii*	UWBM 7227	[45]	[45]	[45]	515
*Phrynosoma goodei*	CAS 229922	[45]	[45]	[45]	574
*Phrynosoma hernandesi*	MVZ 245875	[45]	[45]	[45]	573
*Phrynosoma mcallii*	CAS 229923	[45]	[45]	[45]	538
*Phrynosoma modestum*	MVZ 238583	[45]	[45]	[45]	545
*Phrynosoma orbiculare*	UWBM 7285	[45]	[45]	[45]	508
*Phrynosoma platyrhinos*	MVZ 161495	[45]	[45]	[45]	563
*Phrynosoma sherbrookei*	MZFC 28101	[13]	[13]	[13]	579
*Phrynosoma solare*	MVZ 241510	[45]	[45]	[45]	410
*Phrynosoma taurus*	UWBM 7296	[45]	[45]	[45]	559
Iguanidae outgroups					
*Gambelia wislizenii*	UWBM 7353	[13]	[13]	[13]	549
*Liolaemus darwinii*	LJAMM-CNP 14634	[13]	[13]	[13]	581

Data on species diversity was taken from the Reptile Database [85]

Targeted sequence capture data for species used in previous studies [13, 45] are listed with their voucher information and the number of loci

In order to try to resolve the *Sceloporus* phylogeny and understand the relationship between chromosome evolution and diversification we sought near complete taxon sampling and a broad sampling of loci from throughout the genome. We estimated a phylogenomic tree for *Sceloporus* using targeted sequence capture data that includes a combination of ultraconserved elements [42] and protein-coding genes used in previous studies of squamate phylogeny [43]. These new data are analyzed using concatenation and coalescent-based species tree inference methods. We conduct a diversification analysis to estimate the number of rate shifts and their locations on the phylogeny. These patterns of diversification are then discussed in relation to chromosomal diversity. The results suggest that differentiation in the fundamental number of chromosomes among species groups may be linked to *Sceloporus* diversification.

## Results

### Targeted sequence capture data

**Fig. 1:**
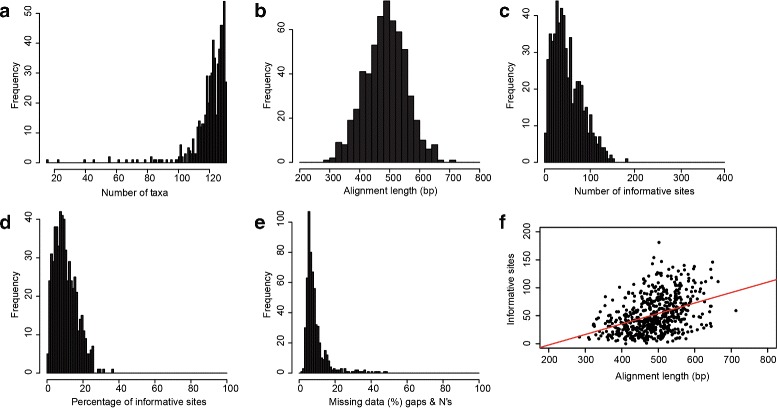
Properties of the targeted sequence capture data collected for phrynosomatid lizards. Frequency distributions summarize the properties of the phylogenomic data on a per locus basis, including number of taxa (**a**), alignment length (**b**), number of informative sites (**c**), percentage of informative sites (**d**), and missing data including both gaps and N characters (**e**). There is a positive correlation between alignment length and informative sites, adjusted R^2^ = 0.1523, *p*<0.000 (**f**)

We obtained targeted sequence capture (TSC) data from 44 Squamate Tree of Life (ToL) loci and 541 ultraconserved elements (UCE’s; Table 2). Summaries of the sequence capture loci were generated using scripts available from https://github.com/dportik/Alignment_Assessment [44]. and frequency distributions summarizing the properties of the phylogenomic data on a per locus basis are shown in Fig. 1. Although we included 131 samples in our analysis (129 phrynosomatids and two outgroup species), the final sequence alignments for the Squamate ToL loci contained 118 individuals on average (46 min. – 129 max.), and the UCE alignments contained 121 individuals on average (15 min. – 131 max.). Some of the phylogenomic data were taken from previous studies, including 11 samples from a study of phrynosomatid lizards [13] and 17 samples from a study of the genus *Phrynosoma* [45]. Sequence capture inefficiency during the probe hybridization step and low sequencing effort are two likely reasons for the lack of data for some individuals across loci. A summary of the variation in the TSC data is provided in Table 2. On average, the Squamate ToL loci are longer compared to the UCEs (538 base pairs [bp] vs. 482 bp, respectively), contain more variation (31 % vs. 19 %), and contain more parsimony informative characters (104 vs. 47).

**Table 2:** Summary of the variation in the targeted sequence capture data

Loci	Number of loci	Number of samples	Length (bp)	Variation (%)	PIC
Ultraconserved elements	541	121 (15–131)	482 (284–713)	19 (2–45)	47 (0–146)
Squamate ToL loci	44	118 (46–129)	538 (355–664)	31 (16–57)	104 (48–181)

Values are reported as the mean and minimum-maximum values. PIC refers to parsimony informative characters

### Phylogenetic analysis

**Fig. 2:**
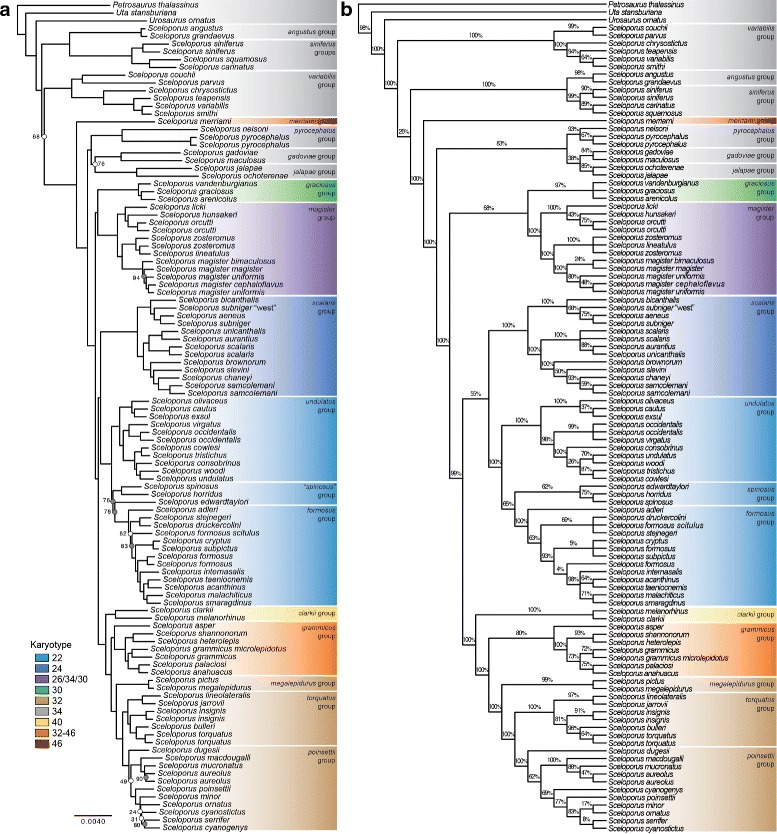
*Sceloporus* phylogeny estimated using targeted sequence capture data. Phylogenetic relationships among *Sceloporus* estimated with concatenation (**a**) and with a coalescent approach (**b**)

The phylogenetic trees that we estimated for *Sceloporus* using the 585 loci using concatenation (RAxML; [46]) and a coalescent-based species tree approach (SVDquartets; [47]) are shown in Fig. 2. The phylogenetic relationships inferred at the base of *Sceloporus* differ between the two approaches. Using concatenation, a clade containing the *angustus* and *siniferus* species groups is sister to the remaining members of *Sceloporus*, whereas in the coalescent tree the *variabilis* group is sister to the rest of the genus. This discrepancy has weak support in the concatenation and coalescent trees (68 and 26 % bootstraps, respectively). The phylogenetic relationships for the remaining species groups are consistent starting at the point in the phylogeny where *S. merriami* diverges. The major relationships include a clade containing the *pyrocephalus*, *gadoviae*, and *jalapae* groups, a clade containing the *graciosus* and *magister* groups, a 22-chromosome clade containing the *undulatus*, *formosus*, and *spinosus* groups (sister to the *scalaris* group), and a 32-chromosome clade containing the *megalepidurus*, *torquatus*, and *poinsettii* groups (sister to the *grammicus* group and the *clarkii* group). The support for these clades varies between the concatenation tree (these relationships all have high support) and the coalescent tree (only the 22 and 32 chromosome clades have significant support). One notable difference is that the concatenation tree fails to support the monophyly of the *spinosus* group, whereas the coalescent tree provides weak support (62 % bootstrap) for this group.

**Fig. 3:**
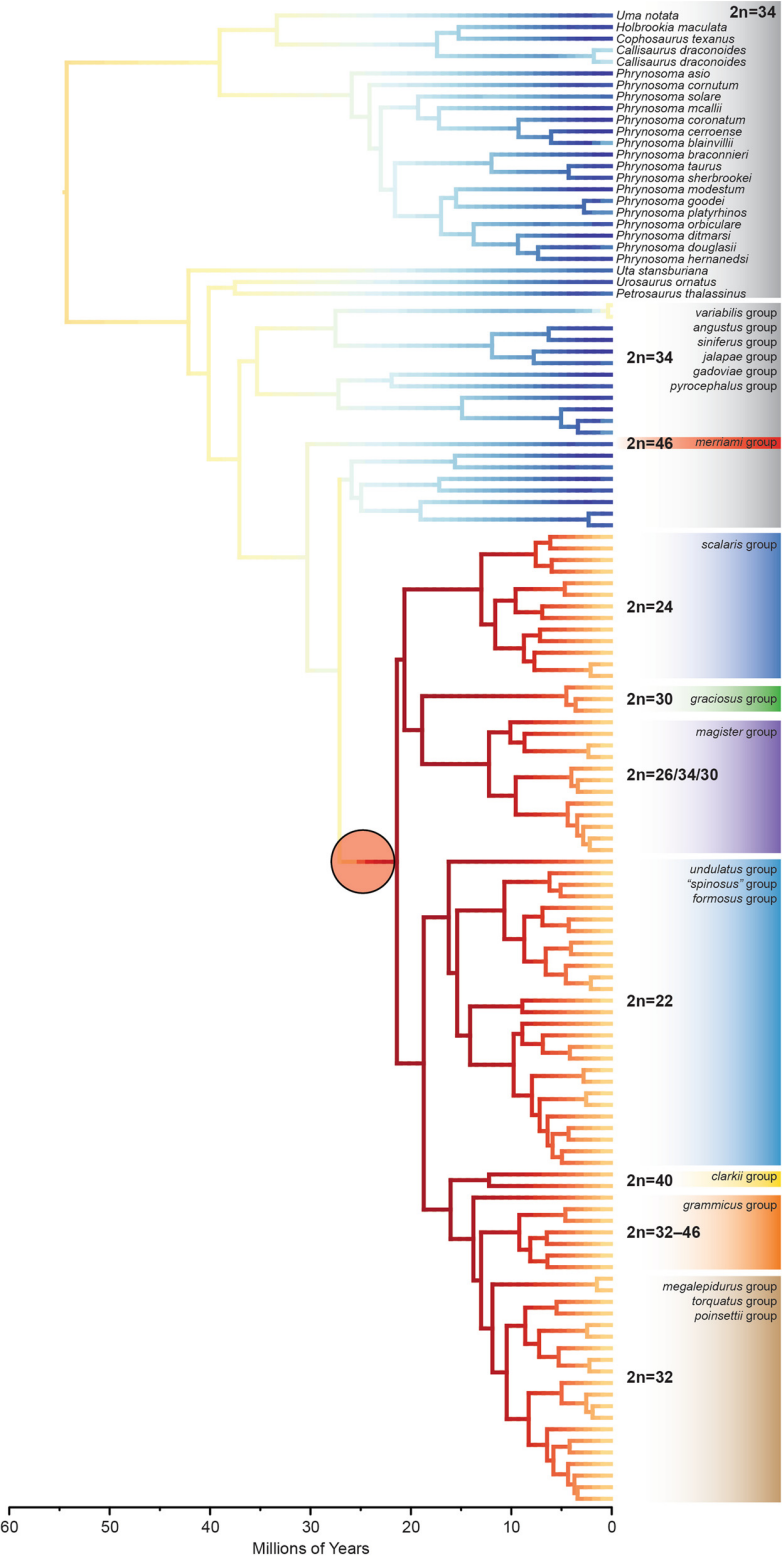
The BAMM analysis supports a single rate shift in *Sceloporus* that coincides with the rapid radiation of species groups containing different numbers of chromosomes

Our time-calibrated phylogeny estimated using the Squamate ToL loci in BEAST [48] (Fig. 3) indicates that the crown age for the family Phrynosomatidae is approximately 54 million years (mean = 54.12, highest posterior density [HPD] = 46.13–61.65 Ma). The age estimate for the genus *Sceloporus* is 37 million years (mean = 37.02, HPD = 30.71–43.71). Both estimates are consistent with previous estimates [30], but this might not be unexpected given that we used a similar prior. In addition, it is likely that the use of a concatenated data matrix in BEAST is causing divergence time overestimation, and that a species tree approach would provide more accurate estimates. The topology of the BEAST tree is largely similar to the concatenation and coalescent trees shown in Fig. 2, but there are several key differences. First, the BEAST tree places the *scalaris* group sister to the *magister* and *graciosus* groups instead of sister to the 22 chromosome clade. Second, the *spinosus* group is paraphyletic and *S. edwardtaylori* is and placed at the base of the 22-chromosome clade. Third, the *grammicus* group is paraphyletic as a result of moving *S. asper* to the base of a group containing the 32-chromosome clade and the *grammicus* group. These differences in topology are likely the result of excluding the ultraconserved elements from the phylogenetic analysis instead of modeling differences between the phylogenetic methods.

### Gene tree congruence and rapid radiation

**Fig. 4:**
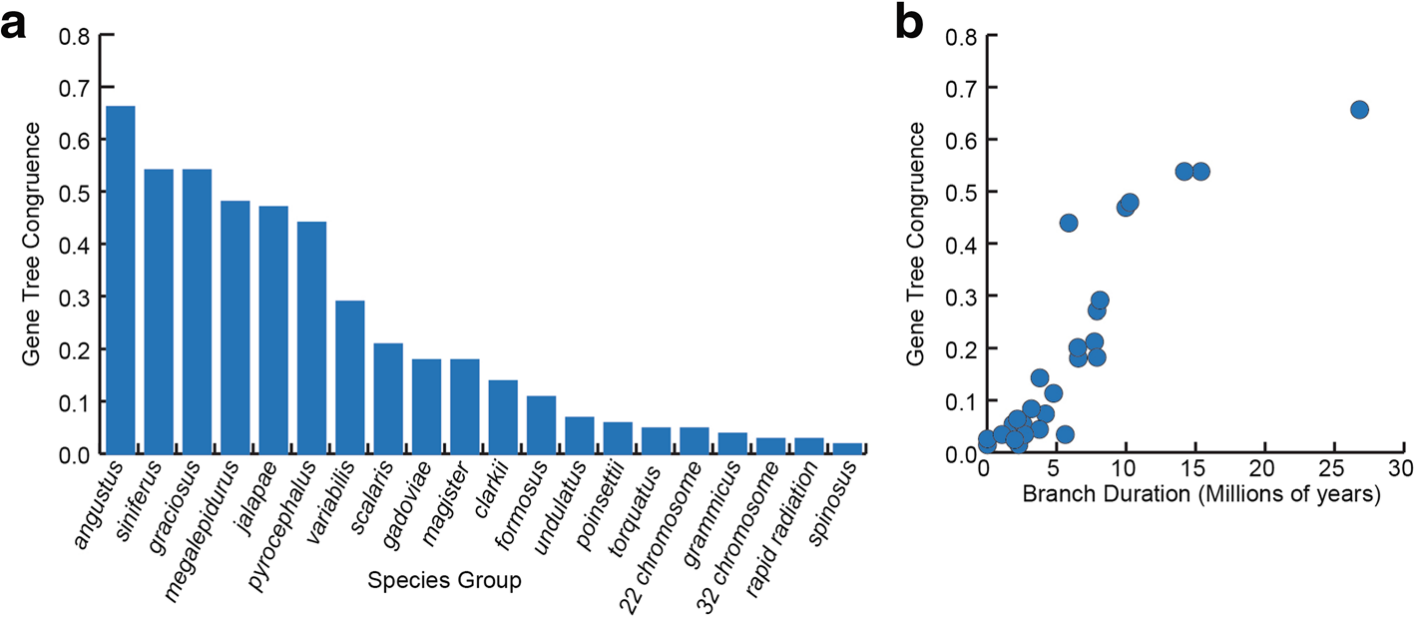
Gene tree congruence in the *Sceloporus* rapid radiation. The majority of species groups in *Sceloporus* are supported by fewer than 50 % of the gene trees (**a**), and there is a positive correlation between gene tree congruence and the duration of a branch (**b**)

Rapid radiations are expected to produce increased gene tree discordance. We investigated congruence between the 585 gene trees (estimated using RAxML) and the estimated species tree by quantifying the number of gene trees that supported the major relationships obtained in the species tree analysis. This approach for measuring congruence does not distinguish between gene tree discordance resulting from a lack of genetic variability versus incomplete lineage sorting. Three *Sceloporus* species groups have gene tree congruence that exceeds 50 % (i.e., at least 50 % of the 585 gene trees support their monophyly): the *angustus*, *siniferus*, and *graciosus* groups (Fig. 4). The remaining species groups have higher levels of gene tree discordance, and some are supported by <10 % of the loci, including the *undulatus* group (40 loci), *poinsettii* group (36 loci), *torquatus* group (30 loci), *grammicus* group (24 loci), and *spinosus* group (9 loci). The 22-chromosome and 32-chromosome clades are supported by 28 and 18 loci, respectively.

There is a strong correlation between the amount of gene tree congruence for a taxon bipartition (e.g., a species group) and the branch length for a taxon bipartition (Fig. 4). We explored this relationship using the branch duration estimates (measured in millions of years) obtained from the BEAST analysis (Fig. 3). As expected, the branches with the shortest time intervals had low gene tree congruence, and branches with longer time intervals had high gene tree congruence.

### Diversification analysis

**Fig. 5:**
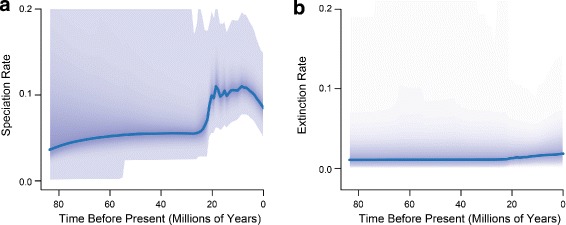
Speciation and extinction rate changes in *Sceloporus*. The speciation rate shift in *Sceloporus* occurred approximately 20–25 million years ago (**a**), but the magnitude of the shift is low (0.05). Extinction rates appear to be constant through time (**b**)

Diversification analyses conducted using BAMM [49] recovered an average speciation rate (*λ*) of 0.09 species/Ma across the phrynosomatid tree. The analysis also found a positive extinction rate (*μ*) of 0.02 species/Ma that has been relatively consistent throughout the history of phrynosomatids (Fig. 5). We found strong evidence for heterogeneous diversification dynamics with a single acceleration in speciation rate at 20–25 million years ago (Fig. 5). The posterior probability for this rate shift occurring on the branch leading to *Sceloporus* species groups exhibiting increased diversity in the fundamental chromosome number is 0.997 (Fig. 3). The following species groups are included in this rapid radiation: *graciosus* and *magister* groups, a 22-chromosome clade containing the *undulatus*, *formosus*, and *spinosus* groups, the *scalaris* group, a 32-chromosome clade containing the *megalepidurus*, *torquatus*, and *poinsettii* groups, and the *grammicus* and *clarkii* groups. Furthermore, we calculated the Bayes factor (BF) for a shift on this branch by incorporating the probability of a rate shift at that branch under the prior alone, and found overwhelming evidence for a shift (BF >139,000). When examined separately, the increased speciation rate for the rapid radiation clade is 0.15 species/Ma, which is double that of the background rate (0.06 species/Ma).

## Discussion

### Chromosome evolution and diversification

The link between chromosomal evolution and diversification in *Sceloporus* has been recognized for decades (reviewed by [24, 31]. A previous study of *Sceloporus* diversification and chromosomal evolution using a Bayesian cross-validation predictive density approach found that species diversity was significantly higher in some parts of the phylogeny than predicted in comparison to background diversification rates [27]. Instead of using a local approach to test hypotheses about diversification rate shifts on pre-specified sections of the phylogeny where chromosomal changes occurred, the BAMM analyses presented here take a global approach with the goal of detecting significant speciation rate shifts anywhere on the phylogeny (Fig. 3; Table 2). The single significant rate shift is estimated to have occurred during the rapid radiation leading to a clade of *Sceloporus* species groups with high diversity in fundamental chromosome number (Fig. 3). The estimated background rates of diversification are similar between the two methods (approximately 0.06 species/Ma), and this rate doubles in the clade containing increased chromosomal diversity (Fig. 5).

Common methods for testing for trait-dependent diversification are the “state speciation and extinction” models (e.g., BiSSE, MuSSE, QuaSSE, etc.) [50]. This family of methods attempts to identify significant speciation or diversification rate differences between species in relation to a trait of interest. This approach sounds appealing for testing the link between chromosome evolution and diversification in *Sceloporus*. However it is important to note that detecting trait-dependent speciation is prone to errors from model violations and model inadequacies, and that these problems have led to an excess of trait-dependent speciation associations in the literature [51, 52]. New statistical tests aimed at distinguishing false associations are available, but these tests are currently limited to binary and continuous characters [53]. In *Sceloporus*, attempting to coerce the multistate karyotype data into a binary model results in few independent associations between the character state and diversification, and this type of problematic character state distribution is expected to return a false positive association [53, 54]. As expected, BiSSE provides strong support for karyotype-dependent diversification in *Scelporus* (results not shown).

Vertebrate radiations, including *Sceloporus*, tend to diversify following a semi-predictable trajectory of divergence [55] along axes of habitat [56], trophic morphology [57], and communication [34, 58–60]. Chromosomal variation is a prominent feature of *Sceloporus* diversity that is putatively linked to their rapid diversification. Disentangling these factors (i.e., ecology, morphology, diet, chromosomes, etc.) to determine their separate and joint contributions to diversification will be an interesting route to take in future studies (see [61] for an example).

Based on a cursory examination of the current geographic distributions of species in relation to their karyotypes, closely-related species of *Sceloporus* with the same karyotype formula are not typically found in sympatry [24]. Instead, communities with multiple species of *Sceloporus* tend to contain species with different karyotypes. The relationship between community assembly and chromosome number has not been formally tested, but we predict that communities of *Sceloporus* will be over-dispersed on the phylogeny and support the observation that species with similar karyotypes are typically not sympatric.

The ancestral karyotype for phrynosomatid lizards is 2n = 34 (12 macrochromosomes, 20 microchromosomes, and an XY sex chromosome pair), and only *Sceloporus* shows variation around this karyotype formula, which ranges from 2n = 22 to 2n = 46 [31]. The speciation rate shift that we detected on the phylogeny (Fig. 3) is located at the base of a clade containing high chromosome number diversity. There are changes in the karyotypes of *Sceloporus* that are not associated with this particular clade, including minor modifications such as inversions and/or secondary constrictions near the centromeres of the macrochromosomes [27]. The most dramatic example of a chromosomal change in a species that is outside of the rapid radiation is *Sceloporus merriami*, which has a karyotype formula of 2n = 46 resulting from the fission of 6 macrochromosomes. The chromosomal changes observed in the species/species groups falling outside of the rapid radiation do not appear to be correlated with any significant shift in speciation rate.

The evolutionary changes in autosomes and sex chromosomes that have produced karyotypic diversity that is distinctive from the ancestral 2n = 34 formula require a reevaluation on our new phylogeny (Figs. 2 and 3). Previous studies suggesting that the *magister* and *graciosus* groups were not sister taxa assumed that they must have independently evolved several unique karyotype features. These groups each have missing or indistinct sex chromosomes, and they each contain 2n = 30 chromosomes (although the *magister* group also contains species with other arrangements). The new phylogenetic trees presented here support these species groups as sister taxa, and therefore the presence of indistinct or missing sex chromosomes presumably evolved once in the common ancestor, and the ancestral karyotype is most likely 2n = 30. The sister group relationship between the 22-chromosome clade and the 2n = 24 *scalaris* group is unchanged (this clade received 100 % support from concatenation, but only 55 % support from coalescent analysis), and this further supports the notion that multiple fusion events are responsible for progressively reducing the number of chromosomes in these groups. The new phylogeny also supports a 32-chromosome clade composed of the *torquatus* group, *poinsettii* group, and *megalepidurus* group. The 32-chromosome clade is sister to the *grammicus* group (2n = 32 – 46), and this clade is sister to the *clarkii* group (2n = 40).

### Resolving rapid radiations

Resolving rapid radiations using molecular phylogenetic techniques requires sequencing a very large number of nucleotides. However, there is an important distinction between obtaining enough nucleotides to resolve a gene tree versus sequencing enough loci to resolve a species tree. Resolving a gene tree should be feasible if enough nucleotides are available at the locus and as long as the rate of evolution is adequate for the scope of the investigation. The extreme of this approach is taken when full sequences are obtained for non-recombining animal mitochondrial DNA (mtDNA) genomes (e.g., amphibians [62], birds [63], mammals [64]) or plant chloroplast genomes [65]. The gene trees estimated from these studies typically provide strong support for phylogenetic relationships, even for species involved in rapid radiations. Despite the strong appeal of obtaining a robust tree from just a single locus, there are many reasons to be suspicious of the relationships in gene trees, including problems associated with incomplete lineage sorting, gene duplication and extinction, and horizontal gene transfer [66], as well as issues related to inaccurate phylogenetic model assumptions (reviewed by [67]). The advantage of sampling independent loci from across the genome, rather than focusing effort on obtaining long sequences from one or a few loci, is that some of these problems can be circumvented in an attempt to obtain a more accurate phylogeny.

In *Sceloporus* lizards, previous studies using mtDNA obtained a fairly well-resolved and strongly supported phylogeny [29], but large discrepancies in relationships were apparent in comparison to a species tree estimated from a few nuclear loci, presumably as a consequence of mtDNA introgression [28]. Instead of sequencing more mtDNA aimed at obtaining an even more robust mtDNA gene tree, we leveraged our resources towards obtaining a large number of independent loci from across the genome using a targeted sequence capture approach. Not all of these loci that we selected were particularly useful for resolving the rapid radiation in *Sceloporus*. Only 3 % of the 585 loci that we obtained supported the rapid radiation that corresponds to the period of increased chromosomal diversification in *Sceloporus* (Fig. 4). The lizard-specific probe set that we designed for this project appears to have been barely capable of resolving this rapid radiation, and it is likely that this same set of markers will be incapable of resolving more difficult phylogenetic problems. Aside from developing a new probe set that targets more loci, two ways to increase the percentage of loci that contribute useful phylogenetic information in a targeted sequence capture experiment are to invest in longer sequence reads and/or optimize the lab protocol to obtain longer loci. Overall, the new paradigm of sequencing 100s or 1000s of loci in order to obtain a few loci that resolve a rapid radiation seems highly inefficient. Developing more refined locus selection methods that can identify loci with optimal evolutionary rates for a specific question, and thereby increase the probability that a loci will contribute useful phylogenetic signal, is an important direction for the future of phylogenomic studies.

### Systematics of *Sceloporus*

The phylogenomic estimates for *Sceloporus* obtained using 585 loci (Fig. 2) provide strong support for relationships that have been difficult to elucidate using smaller amounts of data. At a higher taxonomic level, we find strong support for relationships among genera in the Sceloporine (i.e., [[[*Urosaurus* + *Sceloporus*],*Uta*,],*Petrosaurus*]) that are consistent with a recent study using the same data [13], and restriction site associated DNA sequencing (RADseq) data [68], but conflict with previous estimates that combine mtDNA and nuclear genes [29]. Within *Sceloporus*, the relationships at the base of the phylogeny are weak and differ depending on the analysis type (e.g., concatenation vs. coalescent analysis). More data may be necessary to obtain a definitive placement for the initial divergences within the genus. The composition of the early diverging groups is clear [69], including the *variabilits* group and the close relationships between the *siniferus*, *angustus*, and *utiformis* groups (this group was not sampled in our study), and determining which was the first to diverge requires further study.

The addition of loci has helped provide strong support for some species groups relationships that were unresolved with smaller nuclear datasets. For example, the clade containing the *pyrocephalus*, *gadoviae*, and *jalapae* groups that we obtained with the TSC data is also supported by analyses of mtDNA [28]. However, previous analyses of smaller nuclear gene datasets did not support the monophyly of this group [28, 29]. Several species group relationships have been difficult to determine because of the influence of gene tree conflict between nuclear and mtDNA [28]. One example of this problem pertains to the relationship between *S. clarkii* and *S. melanorhinus*, which differs between mtDNA and nuclear genes [28]. The mtDNA gene tree separates these species across the phylogeny, whereas analyses of nuclear data support them as a clade. We find strong support for a clade containing *S. clarkii* and *S. melanorhinus*, and since species groups are intended to provide names for monophyletic groups, we recommend naming this clade the *clarkii* species group.

We find strong support for a clade containing the *poinsettii* and *torquatus* groups, and we recommend referring to all species included in these groups as members of the *torquatus* species group. The *poinsettii* group was erected to deal with non-monophyly of the *torquatus* group in relation to the *megalepidurus* group [29]. Given that there is no longer any evidence of paraphyly in the *torquatus* group it does not seem necessary to retain the *poinsettii* group.

Monophyly of the *spinosus* group is weak or missing depending on the type of analysis and source of molecular data. A recent phylogeographic study of this species group revealed mtDNA introgression and gene flow between species [38]. These processes are likely responsible for the discordant phylogenetic relationships that have been described for these species [28, 29]. Gene flow and introgression play a prominent role in the evolution of *Sceloporus* [28, 70–72], and future phylogenetic studies of the group will benefit from new analytical approaches that can identify gene flow during species tree estimation.

## Methods

### Specimen collection

The family Phrynosomatidae is a diverse group of lizards with a broad North American distribution from Canada to Panama. Much of their diversity is centered in the arid regions of the southwestern United States and Mexico. The group has approximately 148 species arranged into nine genera. We sampled 129 phrynosomatid individuals, including one sample of *Cophosaurus*, *Holbrookia*, *Petrosaurus*, *Uma*, and *Uta*, two specimens of *Callisaurus*, all 17 species of *Phrynosoma*, and 86 species of *Sceloporus* (see Table 1 for voucher details). We sampled all species groups within *Sceloporus* with the exception of *S. utiformis*. We used *Gambelia wislizenii* and *Liolaemus darwinii* to root the tree. Specimens collected for this project from Mexico and the United States are deposited at the Burke Museum of Natural History and Culture at the University of Washington and the Museo de Zoologia “Alfonso L. Herrera” at the Universidad Nacional Autónoma de México. Specimens were collected with approval from the University of Washington Institutional Animal Care and Use Committee (IACUC #4209-01). Scientific specimens were collected in México with permission from the Secretariat of Environment and Natural Resources (SEMARNAT Permit No. 05034/11 to ADL, and Permit No. FAUT 0093 to ANMO). We also obtained tissue and/or DNA loans from the following genetic resource collections and herpetology collections: Museum of Vertebrate Zoology (University of California, Berkeley), Burke Museum of Natural History and Culture (University of Washington), California Academy of Sciences, Ambrose Monell Cryo Collection (American Museum of Natural History), Los Angeles County Museum, Royal Ontario Museum, University of Texas at Arlington, and the Museo de Zoologia “Alfonso L. Herrera” (Universidad Nacional Autónoma de México).

### Targeted sequence capture data

We collected targeted sequence capture data using a set of RNA probes specific for iguanian lizards (Leaché et al., 2015). We synthesized custom probes that target 585 loci (2X tiling; two 120 bp probes per locus) using the MYbaits target enrichment kit (MYcroarray Inc., Ann Arbor, MI, USA). The probes target 541 ultraconserved elements (UCEs) used in the Tetrapods-UCE-5Kv1 probes (ultraconserved.org; [42]) and 44 nuclear loci used for the Squamate ToL project [43].

Whole genomic DNA was extracted from tissues using a NaCl extraction method [73]. Genomic DNA (400 ng) was sonicated to a target peak of 400 bp using a Bioruptor Pico (Diagenode Inc.). Genomic libraries were prepared using the Illumina TruSeq Nano library preparation kit. The samples were hybridized to the RNA-probes in the presence of a blocking mixture composed of forward and reverse compliments of the Illumina TruSeq Nano Adapters, with inosines in place of the indices, as well as chicken blocking mix (Chicken Hybloc, Applied Genetics Lab Inc.) and salmon blocking mix to reduce repetitive DNA binding to beads. Libraries were incubated with the RNA probes for 24 hours at 65 °C. Post-hybridized libraries were enriched using TruSeq adapter primers with Phusion^®;^ High-Fidelity DNA Polymerase (New England Biolabs Inc.) for 20 cycles. Enriched libraries were cleaned with AMPure XP beads. We quantified enriched libraries using qPCR (Applied Biosystems Inc.) with primers targeting five loci mapping to different chromosomes in the Anolis genome. Library quality was verified using an Agilent Tape-station 2200 (Agilent Technologies). These samples were pooled in equimolar ratios and sequenced using an Illumina HiSeq2000 at the QB3 facility at UC Berkeley.

### Bioinformatics

The raw DNA sequence reads were demultiplexed based on unique sequence tags using Casava (Illumina). We removed low-quality reads, trimmed low-quality ends, and removed adapter sequences using Trimmomatic [74]. The clean reads were assembled for each species using the de novo assembler IDBA [75]. We ran IDBA iteratively over k-mer values from 50 to 90 with a step length of 10. We used phyluce [42] to assemble loci across species. We performed multiple sequence alignments for each locus using MAFFT [76], and we trimmed long ragged-ends to reduce missing or incomplete data. The final data assemblies are often fragmentary as a result of poor sequence quality and/or lack of sequencing coverage across a locus. As a result, sequence alignments tend to contain large regions of gaps separating relatively few nucleotides. Checking large numbers of loci by eye for these artifacts is difficult, and there is a need for the development of new bioinformatic tools that can help increase the accuracy of sequence alignments obtained from large phylogenomic datasets.

### Phylogenetic analysis

We estimated phylogenetic trees using concatenation and coalescent-based species tree inference. For the concatenation analyses, we conducted unpartitioned maximum likelihood (ML) analyses with RAxML v8.0.2 [46]. We used the GTRGAMMA model, and branch support was estimated using 1000 bootstrap replicates. The RAxML tree was rooted with *Gambelia wislizenii* and *Liolaemus darwinii*. We estimated a species tree using SVDquartets [47], a coalescent-based species tree inference method that uses the full sequence data. This method infers the topology among randomly sampled quartets of species using a coalescent model, and then a quartet method is used to assemble the randomly sampled quartets into a species tree. Reducing the species tree inference problem into quartets makes the analysis of large numbers of loci feasible. We randomly sampled 100,000 quartets from the data matrix, and used the program Quartet MaxCut v.2.1.0 [77] to infer a species tree from the sampled quartets. We measured uncertainty in relationships using nonparametric bootstrapping with 100 replicates. The bootstrap values were mapped to the species tree estimated from the original data matrix using SumTrees v.3.3.1 [78].

Divergence times were estimated using BEAST v1.8 [48] using the Squamate ToL loci. The ultraconserved elements were removed from the analysis to help decrease the computation time. We assigned an uncorrelated lognormal relaxed clock site model and use a single calibration with a normal distribution of 55 ± 4 mya over the family Phrynosomatidae [30]. We ran two analyses, one with a Yule (birth only) tree prior and one with a birth-death prior, using random starting trees [79, 80]. The concatenated dataset was run for 10 million generations under the GTR+I+ *Γ* model with four gamma categories. We sampled every 1000 generations and discarded the first 25 % as burnin. Convergence statistics were examined using Tracer v1.6 [81], and assumed to have been met when effective sample sizes (ESS) were greater than 200 for all statistics. We used TreeAnnotator v1.8 to produce the maximum clade credibility (MCC) tree from all post-burnin trees and the 95 % highest posterior density (HPD) for each node.

A prediction from rapid radiations is that gene tree discordance will be high. To investigate the level of congruence between the 585 gene trees and the estimated species tree, we quantified the number of gene trees that supported the major relationships obtained in the species tree analysis. First, we estimated phylogenetic trees for each locus separately using RAxML with the HKY model [82]. Next, we used PAUP v4.0b10 [83] to quantify the number of loci that supported taxon bipartitions that were present in the MCC tree. The taxon bipartitions of interest included the *Sceloporus* species groups and all of the relationships along the backbone of the *Sceloporus* phylogeny. Each taxon bipartition was loaded into PAUP as a monophyly constraint prior to loading the gene tree. Species that were absent from a gene tree were removed from the monophyly constraint. We tallied the total number of gene trees (out of 585 total) that supported each taxon bipartition of interest. Finally, we used the branch duration estimates (in millions of years) from the MCC tree to test for a relationship between the number of gene trees supporting a taxon bipartition and the duration of a branch.

### Diversification analysis

We tested for shifts in diversification rate through time using Bayesian Analysis of Macroevolutionary Models (BAMM v.2.1.0 [49]). BAMM models speciation and extinction rates by simulating rate shift configurations using reversible-jump Markov chain Monte Carlo (rjMCMC). This approach relaxes the assumption of time-homogeneous diversification and allows a vast space of candidate models to be explored [49]. We ran BAMM for 10 million generations on the MCC tree, sampling parameters every 1000 generations. BAMM incorporates incomplete taxon sampling into the likelihood equation; we assume to be missing 20 % of species diversity for the famliy. We used default prior settings, though results are generally robust to the choice of prior under a compound Poisson process [49]. We assessed convergence by computing the effective sample sizes of the log-likelihoods and number of evolutionary rate shifts in R using the package coda [84].

### Availability of supporting data

The data set supporting the results of this article are available in Additional file 1. Sequence reads can be accessed through GenBank under the Accession Numbers KU765209–KU820629.

## Additional file

Additional file 1 Sequence alignment and phylogenetic trees. Full alignment of the targeted sequence capture data (541 ultraconserved elements and 44 Squamate ToL loci) with data partitions in NEXUS format. The file is compatible with multiple programs; it can be imported into BEAUti and exported in XML format for BEAST [48], executed in PAUP [83], or viewed with a text editor. Phylogenetic trees are embedded at the top of the file; these can be copied to a new file and viewed in FigTree. (ZIP 9751 kb)

## Abbreviations

BF: bayes factor
bp: base pairs
HPD: highest posterior density
Ma: million years
MCC: maximum clade credibility
ML: maximum likelihood
mtDNA: mitochondrial DNA
RADseq: restriction site associated DNA sequencing
rjMCMC: reversible-jump Markov chain Monte Carlo
ToL: Tree of Life
TSC: targeted sequence capture
UCE: ultraconserved elements
